# Hydrogeochemistry of seasonal variation of Urmia Salt Lake, Iran

**DOI:** 10.1186/1746-1448-2-9

**Published:** 2006-07-11

**Authors:** Samad Alipour

**Affiliations:** 1 University of Urmia, P.O. Box 165, Urmia, Iran

## Abstract

Urmia Lake has been designated as an international park by the United Nations. The lake occupies a 5700 km2 depression in northwestern Iran. Thirteen permanent rivers flow into the lake. Water level in the lake has been decreased 3.5 m in the last decade due to a shortage of precipitation and progressively dry climate. Geologically the lake basin is considered to be a graben of tectonic origin. Na, K, Ca, Li and Mg are the main cations with Cl, SO4, and HCO3 as the main anions. F & Br are the other main elements in the lake. A causeway crossing the lake is under construction, which may affect the lake's annual geochemistry. The main object of this project is mainly to consider the potential of K-mineral production along with ongoing salt production.

Seven hundred and four samples were taken and partially analyzed for the main cations and anions. Surface water (0.5 m. depth) was analyzed for Na, K, Mg, Ca, Br and Li, and averaged 87.118 g/lit, 1.48 g/lit, 4.82 g/lit, 4.54 g/lit, 1.19 ppm and 12.7 ppm respectively for the western half of the lake. Sodium ranged between 84 to 91.2 g/lit, and showed higher concentrations in the south than in the north. This unexpected result may be caused by shallower depth in the south and a higher net evaporation effect. Calcium ranged between 4.2 to 5 g/lit, apparently slightly higher in the north. K is higher in the south, possibly due to rivers entering from south that may carry slightly higher K in solution.

In the middle-range samples (0.5–5 m.), K averaged 1.43 g/lit and ranged from 1.40 to 1.46 g/lit. At this intermediate depth the distribution of K is clearly higher to the south of the causeway that is currently under construction. It is not clear whether this increase is the effect of the causeway or the effect of the salty Aji-Chay River to the east, and the Khoy salt domes to the north of the lake. At depth (5 m–10 m), K averaged 1.48 g/lit and ranged from 1.4 to 1.49 g/lit, differing only in the second decimal from the average of the middle and surface samples.

Ignoring the small difference between the averages of the three sample depths, the distribution of K is highly homogeneous in the lake water due to the mixing process. Therefore causeway construction has not yet strongly affected K distribution, or it may be at the starting point. Magnesium concentration ranged from 4.6 to 5-g/lit, and was elevated in the south. This differs somewhat compared to calcium. Lithium, with an average of 12–13 ppm, is slightly higher in the south, and has not shown any significant variation in all three seasons. Iodine was below the detection limit in the lake.

Urmia Lake, geochemically, is highly uniform both to the south and north of the causeway, in both the surface and deep brines. K and Mg, which average 1.48 and 6.6 g/lit in order, could be elements worth production in addition to the NaCl currently being produced from the lake. Br, F, Li and B in the limit of <50 ppm don't look to be in the economical range.

## Background

Urmia Lake, due to its unique characters, is designated as an international park and protected biosphere reserve by the United Nations [1]. Also, there is a strong tendency by the Iranian Environmental Protection Organization to protect the lake's environment. At the same time, increased demands to exploit salt and other elements such as K, Li, Br, and some other elements, especially by private sectors, makes it necessary to understand the nature of the lake and its chemical composition at different depths and in different areas. At present, 450,000 tones of salt is exploited annually (figure 2). Four hundred thousand tons are used for the production of NaCO3 in Maragheh city at the southwest corner of the Urmia Lake, and the remaining 50,000 tons is produced by small private sectors for export and for domestic uses in villages around the lake. This research, compared to the previous works, is the first systematic regional chemical study which has never been conducted in the lake, and is designed to study the seasonal changes in chemistry with the main emphasis on the potential production of K. Potassium is now produced from some playas such as Gawkhoni playa at central Iran [2].

**Figure 1:**
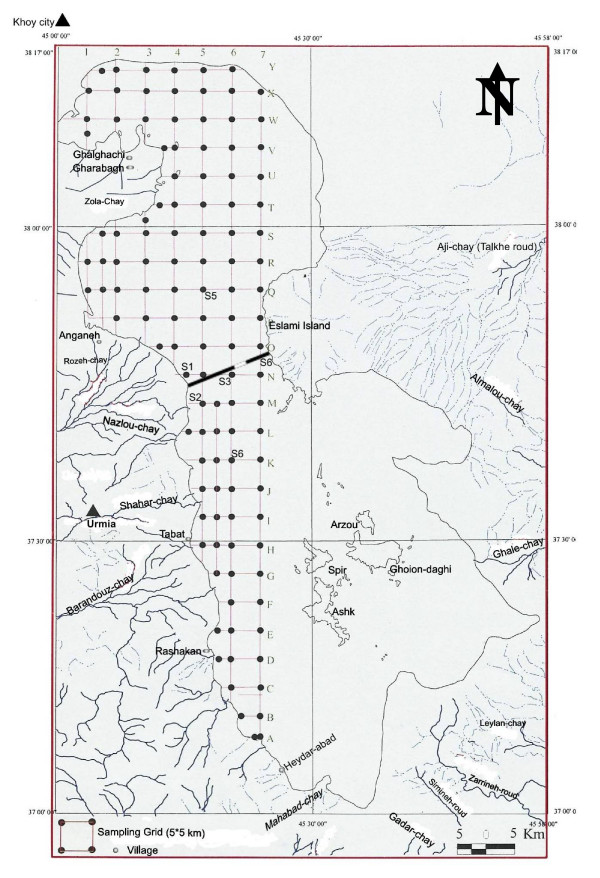
Sampling grids and the rivers flowing into the lake.

**Figure 2:**
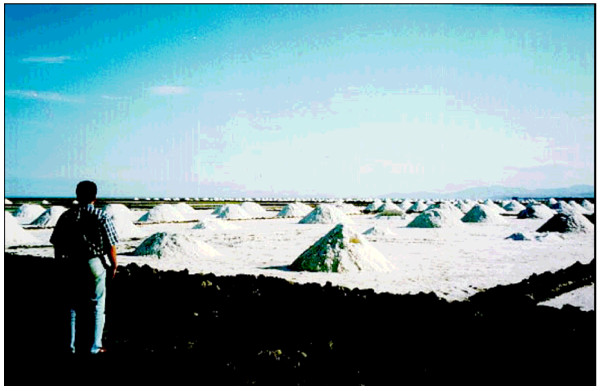
Salt harvesting from Urmia Lake.

## Methodology

### Sampling

#### Orientation sampling

In order to get a preliminary idea about the chemical quality of the lake water, before the main-stage sampling was done, 10 samples from 0.5 meter depth were collected. These samples were analyzed for Mg, Ca, K, Na, SO4, Cl, Br, F, I, Li, B, HCO3, TDS, pH, and Specific Gravity (table 1). Both orientation and the main stage sampling to follow were analyzed in the Pajoheshgaran Chemical Commercial Lab (PCCL) based in Tehran, by the following methods:

**Table 1:** Results of orientation stage analysis: * (g/l), ** (ppm), 15 Oct. 2002

Samples Elements	Salt Pool water	Vs6	Vm6	Vd6	Ks7	Km7	Cs7	Cm7	Ps7	Pm5	Pd5	Average
Mg*	5.7	6.6	6.6	6.7	6.6	6.8	6.7	6.7	6.5	6.5	6.4	6.6
Ca*	1.06	1.22	1.21	1.20	1.22	1.23	1.24	1.20	1.20	1.20	1.0	1.21
K*	0.93	1.11	1.09	1.10	1.10	1.11	1.11	1.12	1.08	1.07	1.08	1.10
Na*	108	88	88	88	87	91	90	91	87	87	87	88
SO_4 _*	11.8	14	14.1	14.1	14.4	14.6	14.6	14.3	13.9	14.1	14	14.2
Cl*	158	153	152	152	153	163	152	153	153	153	152	153
Br**	3.0	1.5	1.5	1.5	1.5	2.2	2.2	1.5	1.5	1.6	1.4	1.6
F**	40	50	45	45	45	50	50	45	45	50	45	47
I**	Nd	Nd	Nd	Nd	Nd	Nd	Nd	Nd	Nd	Nd	Nd	Nd
Li**	15	14	13	13	13	14	14	14	13	14	13	13.5
B**	56	73	76	73	78	80	75	80	75	78	78	77
HCO_3_**	220	285	285	280	280	285	280	285	280	290	290	284
TDS*	330	283	281	284	287	293	280	290	285	288	283	285
pH	6.9	6.7	6.8	6.8	6.7	6.8	6.8	6.7	6.8	6.8	6.7	6.75
	450	390	390	390	395	400	380	400	390	400	390	392
Sp.G	1.21	1.18	1.18	1.18	1.18	1.18	1.19	1.19	1.18	1.18	1.17	1.18

1- K and Na by Flame Photometry

2- Ca and Mg by EDTA Titration

3- Cl by Mohr method

4- B, Br, F, and I by Spectrophotometertery

5- SO4 by Gravimetery

6- HCO3 by volometery (Titration)

7- Other factors by conventional chemical methods.

After the samples were collected, they were kept at the same temperature of sampling time over night and transferred to Tehran the following morning by plane for treatment, filtration, and fixation, etc. The pH of the samples was measured at the sampling sites V6, K7, C7, P7, and P5, where s, m, and d represent surface, middle, and deep (table 1) and from 6 stations, S1 to S6, organized around the causeway (figure 1 & table 2). Computer software such as Excell^®^, Autocade 14^®^, Winsurf^®^, Spss^®^, and Arcview^® ^were used to process and analyze the data.

**Table 2:** PH variation during 6 month from 6 stations (Map 1)

PH/2002–2003	Mar.	Apr.	May	June	July	Aug.	Station average
S1	7.87	7.89	7.98	7.98	7.84	7.35	7.82
S5	7.87	8.05	8.06	8.06	7.92	8.45	8.07
S2	7.92	8.06	8.63	8.07	7.89	7.34	7.99
S6	7.8	8.06	8.11	8.07	7.92	7.34	7.88
S3	7.87	8.07	8.2	8.07	7.9	7.33	7.91
S4	7.88	7.98	8.23	8.06	7.9	7.32	7.90
monthly Lake Average	7.87	8.02	8.20	8.05	7.90	7.52	**7.93**

#### Main sampling stage

In this study, due to limited financial support, only the western half of the lake was covered by systematic sampling, consisting of a 5 × 5 km grid system totaling 115 fixed stations (figure 1). A Magellan^® ^GPS 320 was used to locate each sampling site. All samples were analyzed for the target element "K" as probable economical resources as part of a national program, and due to limited financial support, only part of the samples was analyzed for other elements.

During the main stage sampling, over 653 samples were collected (tables 3, 4 &5) and analyzed in three seasons to include 221 in fall (Oct.–Nov.), 216 in winter (Jan.–Feb.) and 216 in spring (Mar.–Apr.). Samples were taken during 2002–2003 by a Van Dorn^® ^sampler, which could be opened and closed at any selected depth, and thus let the samples be collected without mixing with surrounded water.

**Table 3:** Fall (Oct.–Nov.)

Sample type	No. sample	Analyzed elements
Surface	95	K
	22	K, Na, Li, Mg, Ca, Br
	7	As
Middle	31	K
Bottom	73	K
Total	228	

**Table 4:** Winter (Jan, Feb.)

Sample type	No. Samples	Analyzed Elements
Surface	95	K
	23	K, Na, Mg, Ca, Br, Li
	7	As
Middle	18	K
Bottom	80	K
Total	216	

**Table 5:** Spring (March, April)

Sample kind	No. Sample	Analyzed Elements
Surface	115	K
	23	K, Na, Mg, Ca, Br, Li
	7	As
Middle	12	K
Bottom	66	K
Total	216	

Samples were collected at three levels from the surface to the depth of 9 meters at each station as described below:

(1) Surface samples from 20–50 cm below the water surface (marked by s in the text).

(2) Middle-interval samples from 3–5 m below the surface (marked by m in the text).

(3) Bottom samples taken from depths greater than 5 meters depth or close to the lake bottom (marked by d in the text). Samples collected from all depths and all three seasons, and all other sample types were analyzed for K. Only 68 samples from the three seasons were analyzed for Na, Mg, Ca, Br and Li (22 in autumn, 23 in winter, and 23 in spring).

## Previous studies

During the last two centuries, scientists were interested in understanding the chemical composition of the Urmia Lake, for example: Shlimard [3], Shirokokorof [4]. In 1915, Wag Khlopin [5] studied and analyzed the lake water in the Gharabagh village area for major salts, reporting 34 g/lit NaCl, 5 g/lit Mg compounds, with 138 g/lit of unknown elements. Joneidi has discussed the medical aspects of sediments and salty water of the Lake [6].

In the last 30 years, many researchers have carried out studies on the lake. Just recently, some students have carried out various lake-related studies as their university theses. Unfortunately, most of these studies have had a weak economical look at the lake. Afshar [7] studied the annual change of some chemical parameters to include: EC, TDS, DO, COD, BOD, TOC, and Hg in soil, and surface and underground water of Zarrineh-roud delta south of Urmia Lake. Aazami [8] did some work on water composition at Heidarabd area, southwest of Urmia Lake. Alipour et al., [9] studied the hydrogeochemical composition of soil, surface and underground water of Zarrineh roud delta in the southern part of Urmia Lake. Baveghar et al., [10] investigated the source of salt in Urmia Lake. Daneshgar [1] did some work on water composition in the Sharafkhaneh area, east of Urmia Lake. Ghaheri et al., [11] completed a summary review with limited information on the geochemistry of the lake.

Kariminejad [12], Kaviani [13], Azizi [14] and Kelts et al., [15,16] evaluated resource assessments of Artimia and biomass. Komieli,B., [17], Sedighian et al., [18], and Shahrabi et al., [19,20] investigated chemistry and the engineering geology of the Urmia Lake. Tolouei et al., [21] carried out a limited hydrogeochemical of the lake water, and the Western Azarbayejan Water Bureau has collected information on statistical aspects of the lake and the incoming rivers [22].

Although geology and the resource assessment of the Urmia Lake have been discussed by some others, none have done a systematic comprehensive study of the geochemistry of the lake. Most of previous studies were based on only a few randomly selected samples; therefore the results could not be used to compare with the sampling results of this research.

## Geography

Urmia Lake, occupying 5700 km2 of a depression, is located between western and eastern Azarbayjan territories in the uppermost northwestern part of Iran. Additionally, it is located in 37 – 38 latitudes and 45 – 46 longitudes (figure 1), with altitude of 1250 m. [21]. Based on Landsat data (TM 152 RGB, 1996), the lake's length in the north-south direction varies between 140–144 km and it's width has measured 16 to 63 km (Kelts and Shahrabi [15,16,20].

**Figure 3:**
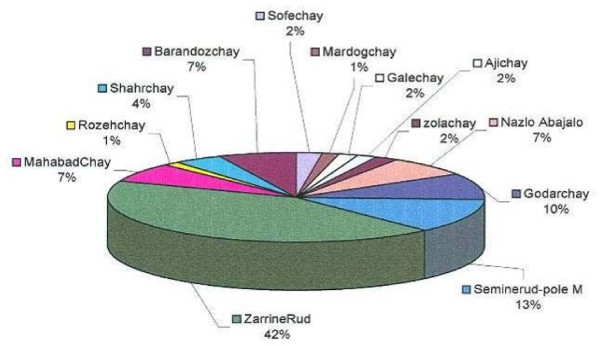
Comparison of rever debits flowing into Urmia Lake during 1991–2000.

**Figure 4:**
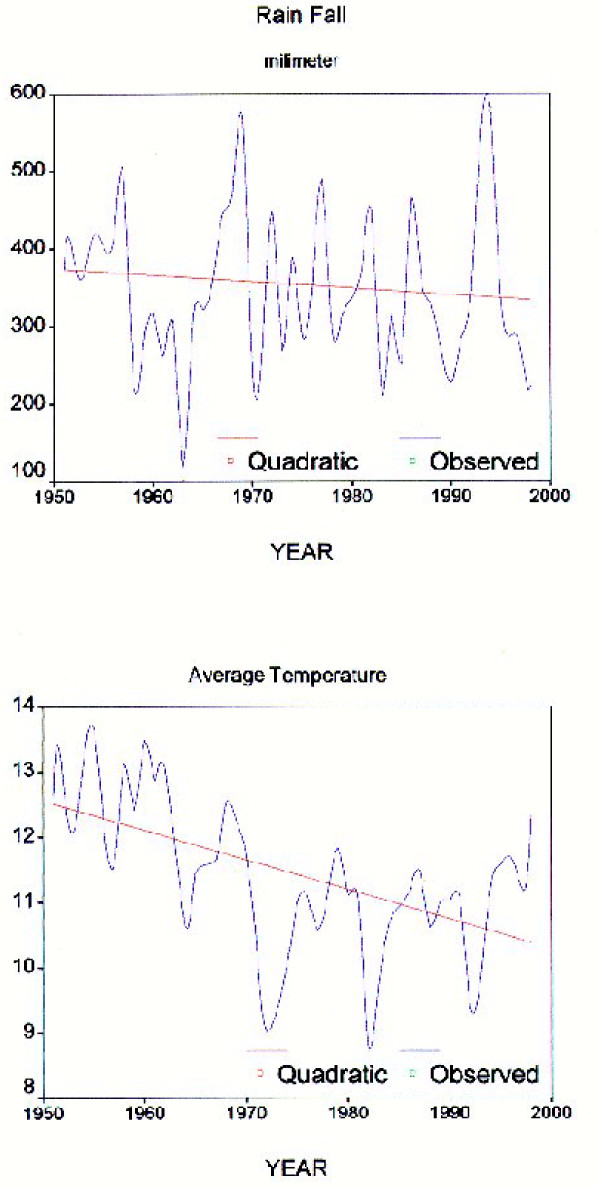
Average rainfall and temperature during 1950–2000.

Thirteen main rivers of various lengths flow into the lake, after crossing many geological formations (figure 1 & figure 3). Due to 10 years of progressive dry climate in the area, the water level is 3 meters less than it was 20 years ago. Precipitation between 1950 – 1998 (figure 4) indicates two cyclic periods of approximately 10 year intervals with high peaks at 1957, 1969 and 1994; and a low in1963.

Historically, the volume of water in the lake was estimated to be 19,000,000,000 M3, with an area of 5700 Km2. The area now has decreased to 4610 Km2 due to dry climate in the last decade [9]. The average annual rain fall in last 28 years is 342 mm and the evaporation at the same period is 1435 mm. So, if the annual river flow added 1093 mm the lake water would be balanced with little change. The sum of river flows in the last 9 years was 138 m3/s, brings 4,351,968,000 m3 of water annually, increasing the lake water level by 0.978 m and resulting in an annual decrease of 32 cm in lake level.

The lake water is clear but its view looks blue and taste slightly bitter but very salty. The annual temperature of the lake area varies between 0 and 20°C in winter up to 30 C in the summer. The overall annual rainfall during 1951–1998 was 347 mm [21], (figure 4).

Urmia Lake is nearly saturated with halite but salt has not been precipitated on the lake floor in the past as indicated by drilling in the vicinity of the highway, except in the summer of 2002 during which salt started to crystallize as a very thin layer (< 1 cm) on the surface of the beach gravels and on the lake floor, but only in the areas with < 2 meters of water depth. This situation produced severe problems for flamingos, ducks and other sea birds feeding from lake Artimia [23]. Salt crystallizes in bird's internal organs, and even their feet were covered by salt balls, which prevented them from walking and flying. Internal crystallization of salt in their internal organs causes mass killing of bird's population in dry seasons [23].

Thirteen main Rivers form the source of water to the lake, to include Zola-chay, Rozeh-chay, Nazlou-chay, Shahar-chay, Barandouz-chay, Gadar-chayr, Mahabad-chay, Simineh-roud, Zarrineh-roud, Leylan-chay, Ghaleh-chay, Almalou-chay Aji-chay (Talkhe-roud), and some other seasonal rivers such as Sofe-chay and Mardog-chay (figures 1 and 3). All of them carry fresh water to the lake except Aji-Chay and seasonal creeks that flow through or pass over the salt domes of the city of Khoy area.

**Figure 5:**
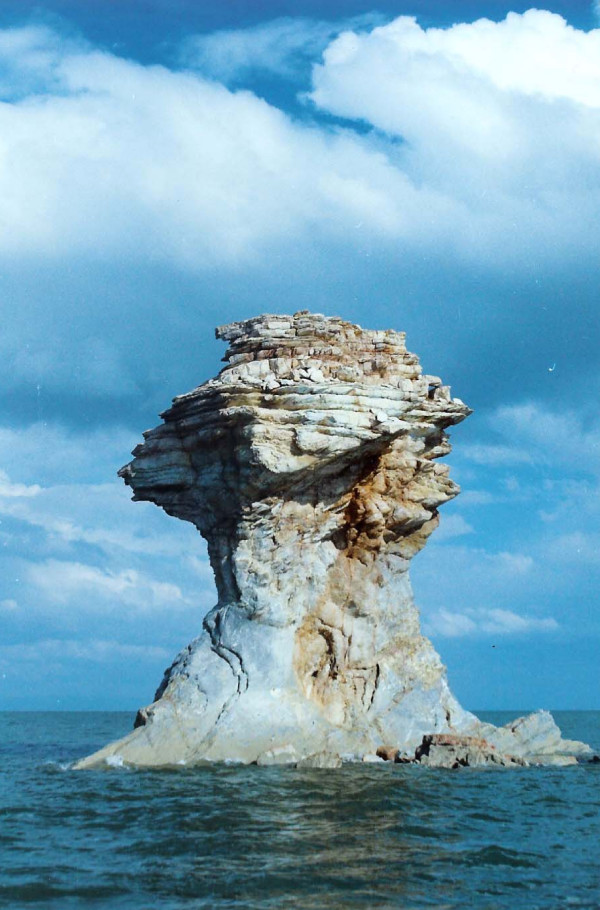
Osman fist, the smallest island of Urmia Lake (height is 10 meters).

One hundred and one islands of various sizes are distributed through the lake. Some of them have substantial area but have very limited fresh water as permanent or seasonal springs. Ghoion Daghi, the largest one, is 6 by 12 Km (figure 1) and the smallest one located in mid-east part of the lake is called Osman feast and is elevated up to 10 meters from water surface (figure 5). Ghoion Daghi has enough fresh water for 16,000 Iranian yellow deer living under strict governmental control [1]. The altitude of Urmia Lake is 1250 meters above sea level. Its length is 114 Km elongated NS ward and 63 Km overall width. The narrowest part is 14 km. The Klantari Causeway, currently under construction, is located at this point forming a dyke of 18 km length and a planed bridge of 1.6 km at middle of the lake to allow for water circulation (figure 1).

**Figure 6:**
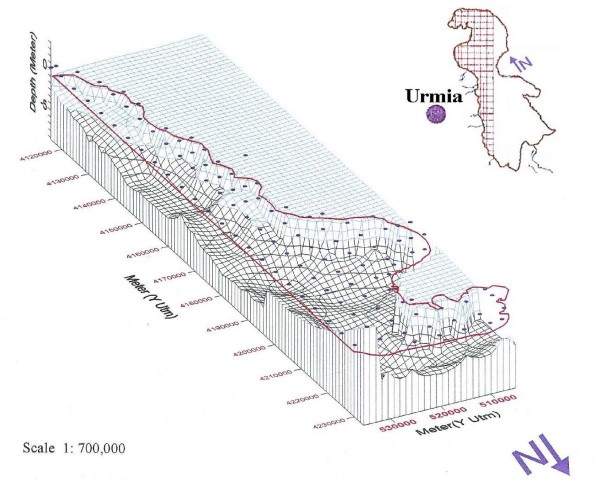
Bathymetric map of western half of Urmia lake.

**Figure 7:**
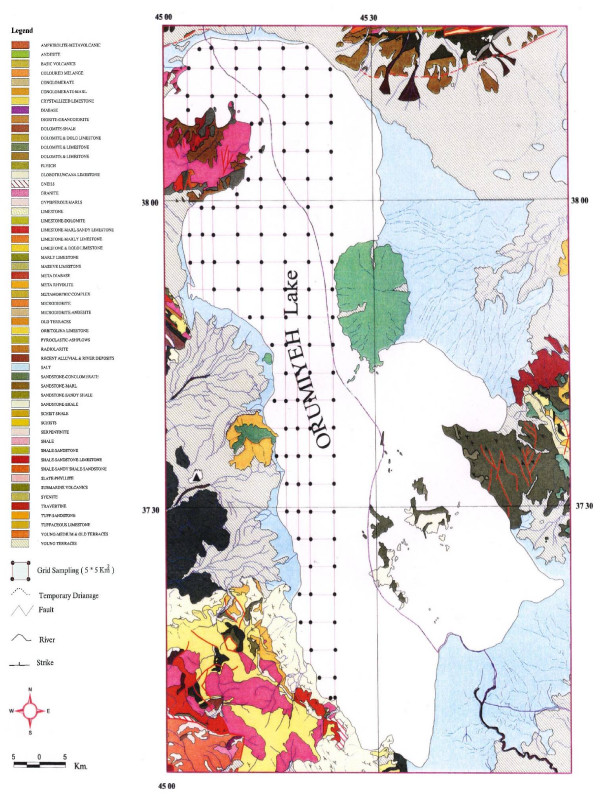
Geological map of Urmia Lake and the surrounding area.

Bathymetry of the lake, determined during sampling, indicates that the depth of the lake in the southern part is shallower, deepening toward the north (figure 7). The deepest point in the sampled areas was about 9 meters; probably it may be a bid deeper in the eastern half near by the vertical banks.

## Geology

Geologically, Urmia Lake is supposed to be a tectonic origin Graben situated among the Saudi Arabia, Urmia, Turkey, and Iran platforms [24]. Highly-elevated mountains of igneous and sedimentary formations surround the Urmia Lake (figure 6). The geology of the area consists of rocks from Pre- Cambrian to Quaternary and very recent lake sediments.

The oldest units of the Pre-Cambrian are formed of meta volcanic series, acidic tuff and diorite in the west, as well as metamorphic amphibolites and gneiss accompanied with under- saturated, alkaline potassic leucit tephrites, leucit basanite, and leucit phenolite. The south shore is occupied by an extensive Zarineh and Simineh Rivers joint delta.

The Paleozoic is represented by dolomite, shale, and sandstone converted by laterite in the late Paleozoic implying a continental environment. Mesozoic rocks crop out in the form of fossiliferous Ammonite limestone and dolomite. Cretaceous rocks in the lake area are extensively thick, forming base conglomerate, sandstone, and limestone. The Ophiolit unit is observed in the west bank and consists of basic and ultra basic rocks with schist and radiolarite limestone recognized by Globotroncana fossils [24].

Tertiary rocks in the lake area are represented by limestone, conglomerate, sandstone and shale converting to tufaceous material with vertebrate fossils from Miocene to quaternary dated 6.9 M.Y. [25]. The most important feature is extensive volcanic activity in the Paleocene-Pleistocene. Old river traces, travertine, lake sediments, and deltaic salty alluvium indicate quaternary in the banks of the lake. From the structural point of view, the Urmia Lake area is located in a zone forming the cross point of Alborz and Zagross ranges [24].

The base of the lake, and most of its islands, consist of cretaceous limestone, Miocene marns, and gently to unfolded limestone (Qum formations) covered by lake sediments dated back to 30,000–40,000 years old. The age of the beginning of the lake basin is reported 400,000 – 800,000 years [17], [19,20], but the age of the modern lake has been calculated from 8000 up to 40,000 years [15]. There are no lake sediments seen at elevations more than 5 meters higher than the present lake level. This may indicate that the lake has never been much larger, and it may have been extended only after a fault, rising up in the north of the lake, pushed the water back towards the south in the graben-like basin, thus forming the modern north-south morphology of the lake.

## Mineralogy

**Figure 8:**
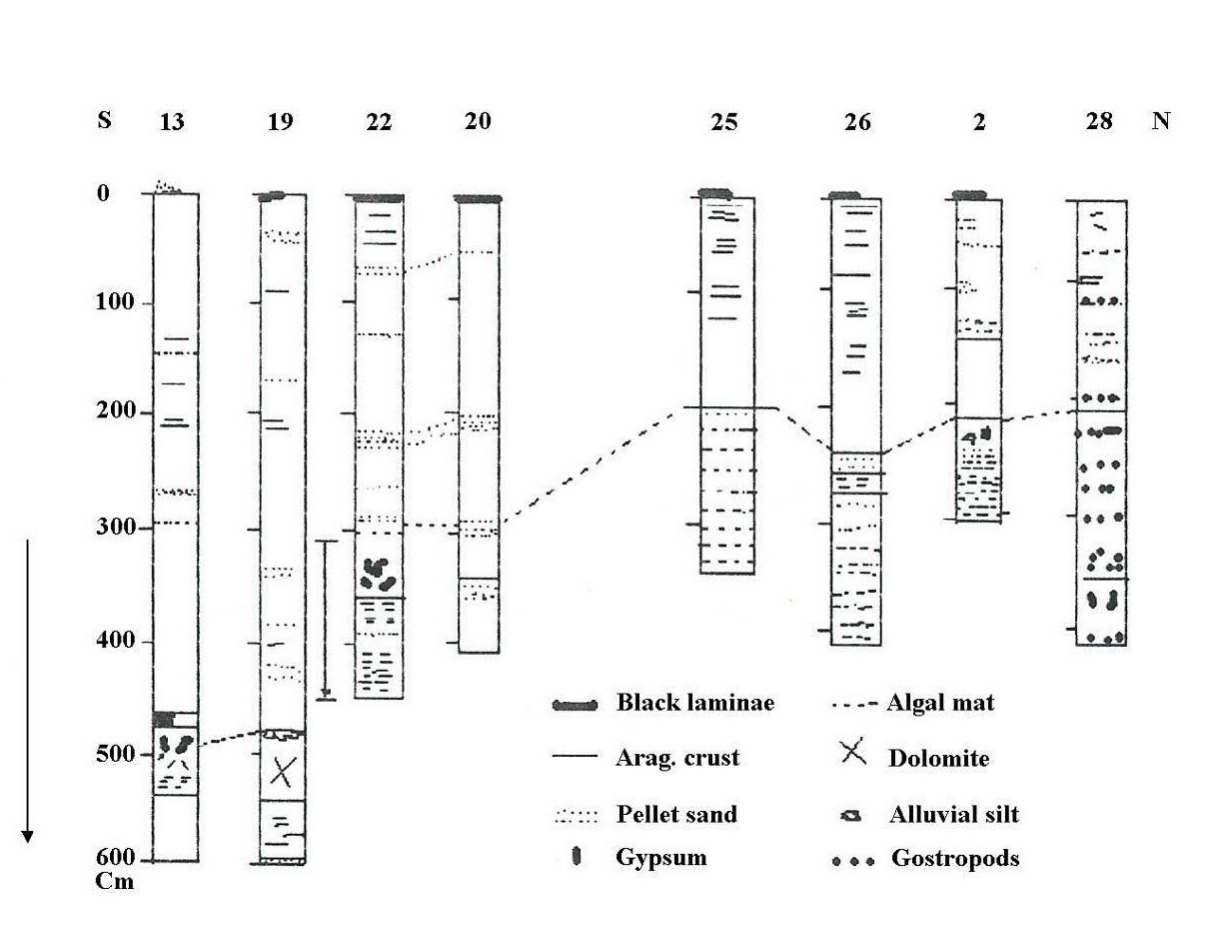
Mineralogy and sediments in drillings of the lake basin (after Kelts, K. and Shahrabi, M. 1986).

From drilling in the lake bed (figure 8) the presence of the following minerals was observed [15]:

1. 1–6 m. of black to green silt and marns containing quartz, calcite, plagioclase and kaolinite.

2. Aragonite is one of the main minerals in the lake bed forming thin, acicular and twin blades (10 microns up to 10 mm). Calcite is another mineral mainly transported by rivers and partially formed in-situ. Calcite increased downward probably indicating low salt at early time of lake formation, and may also indicate an organic source from common green algae.

3. Dolomite identified during the drilling has formed two distinct layers of about 50 cm each of a green color with weak layering in the bottom changing to brown and gray color and blade shape toward the top.

4. Gypsum is observed among the sediments forming thin clean prisms, equant blades up to two mm in length and some coarse-twined gypsum up to 5 cm long. Gypsum formation lowers the Ca and SO4 associated with high K moles. Part of the discussed minerals, concentrated in the lake bed and mixed with algae and detrital minerals; have made black sediments rich in organic materials and 2% carbon. The decay of organic remnants produces unpleasant an odor [15,17]. In the banks, remnants of volcanic rocks, quartz, dolomite, carbonates, and mica form most of the soft, black muddy sediments.

The lake's salty water does not let animals or plants live in it except for **Artimia Urmiana Salina **and some algae species [16,18]. There was no salt bed detected during drilling or salt precipitation in sediments from lake bed up to the base rocks. This indicates that the source of all salt in solution comes from the rivers, that run throught the Khoy salt diapers, and some brought in by the Ajichay river from the east, probably since the date of the lake formation. Although there is not remarkable spring inflow to the lake, there might be some springs in the 50,000 m2 lake bed, but apparently none has been observed or confirmed. The groundwater in the western part of the lake is hypersaline, and very salty in eastern part. Salty water extends to, and occupies more cultivable lands every year, specially, where agricultural water wells in the vicinity are over used.

## Chemical analysis

Ten analyzed orientation samples from 0.5 m. depth showed that Na, K, Ca and Mg are the main cations in the lake (table 1). Na as NaCl was produced from the human age in the area. K and Mg, forming 1.10 g/lit and 6.6 g/lit in order, may have potential for production from the lake. Br, F, Li and B in the limit of <50 ppm don't look to be in the economical range. The unexpected analytical result belongs to iodine, which is below the detection limit in the lake. The lake water's specific gravity varied from 1.18 to 1.21 dependent on overall NaCl in solution. In the main sampling stage for three seasons 704 samples were taken and analyzed for K and partially for other elements as described in the following sections.

## Observations and discussions

### Fall sampling

#### Surface samples

One hundred and twelve surface samples from the 0.5 m depths have been analyzed. The result indicates that K in the surface layer averages 1.45 g/lit, and ranged from 1.39 to 1.5 g/lit. Differences between north and south are not very significant but it looks like K is lower in the north and higher in the south. This slight variation may be due to larger water flow from rivers entering south, possibly carrying slightly higher K in solution, or it may be due to a shortage of water flow from the almost dry drainages from north. North and south are marked by an intermediate zone located immediate south of Kalantary causeway (figure 9.1).

**Figure 9:**
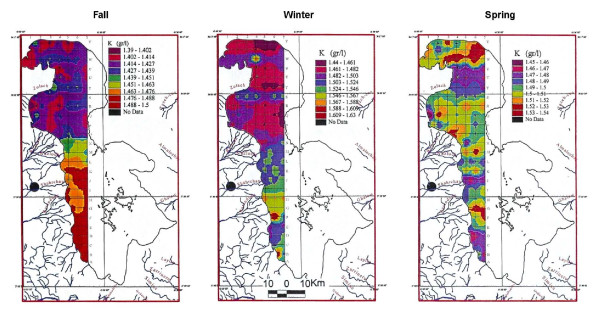
Comparison of potassium in top 0.5 m of Urmia Lake in three seasons.

#### Middle samples

Thirty-one samples analyzed for K from 0.5 to 5 m depths, averaging 1.43 g/lit, ranged from 1.40 to 1.52 g/lit. At this depth interval the distribution of K is clearly higher (figure 10.1) in the south, separated by an intermediate zone, south of Kalantary causeway. It is not clear whether this is the effect of the causeway construction or partially affected by the shortage of water flow from Aji-Chay River and drainages around Khoy salt domes in the west of the lake, during last decade of dry period. Overall, the patterns are similar to the surface samples.

**Figure 10:**
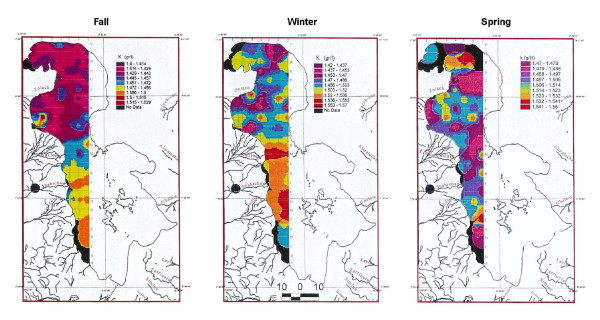
Comparison of potassium in 0.5 to 5 meters of Urmia Lake in three seasons.

#### Bottom samples

Seventy-three samples analyzed for K from 5 to 7 m depths, averaged 1.48 g/lit, and ranged from 1.4 to 1.49 g/lit. K distribution is similar to upper part but inconclusive due to lack of enough data (figure 11.1)

**Figure 11:**
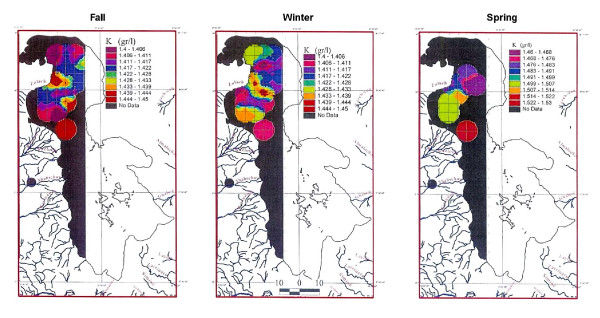
Comparison of potassium in the bottom of Urmia Lake in three seasons.

Ignoring a very small difference between the average of three sample depths, the distribution of K is highly uniform in the lake water. Therefore causeway construction has not yet strongly affected K distribution or it may be just at the starting point.

Twenty-two other samples of surface water for the fall season were analyzed for Ca, Na, Mg, Br and Li averaging 4.54 g/lit, 87.118 g/lit, 4.82 g/lit, 1.19 ppm and 12.7 ppm in order. Sodium ranged between 83.9 to 91.2 g/lit, and showed higher concentration in the south than in the north with an intermediate zone south of the causeway (figure 12.1). This unexpected result, in spite of higher NaCl water entering the lake from the northwest from Khoy salt domes and Aji-chay River from Tabriz area, may be caused by shallower depth in the south and higher evaporation effect in the shallower waters, or it may be due to flow shortage from the north during summer season. Calcium ranged between 4.2 to 5 g/lit, and is apparently slightly higher in the south, decreasing toward the north (figure 13.1). Magnesium concentration ranged between 4.6 to 5-g/lit, and was elevated in the mid-south with slight decreasing toward the north and northeast (figure 14.1). Lithium has not shown any variation in all seasons, and the average of 12–13 ppm is rather constant with only slightly higher concentration in the south (figure 15.1).

**Figure 12:**
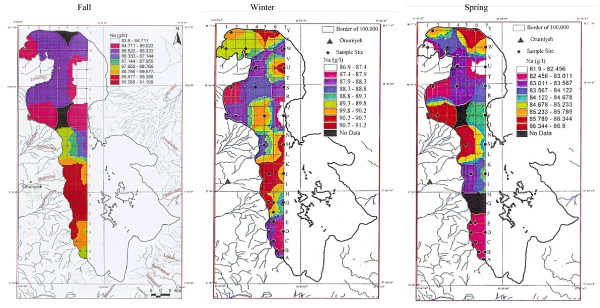
Sodium concentration in the top 0.5 m of Urmia Lake.

**Figure 13:**
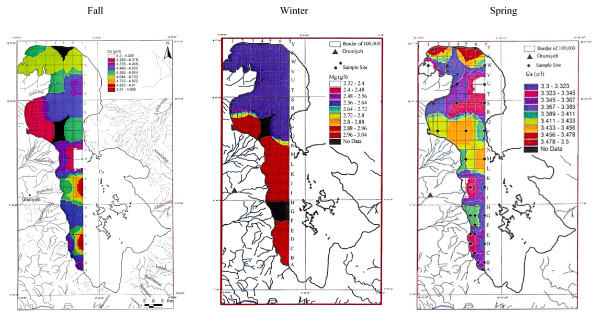
Calcium concentration in the top 0.5 m of Urmia Lake.

**Figure 14:**
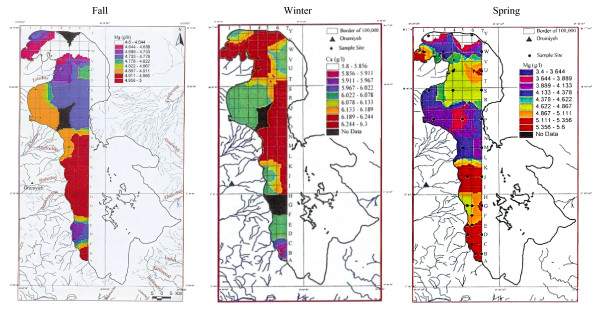
Magnesium concentration in the top 0.5 m of Urmia Lake.

**Figure 15:**
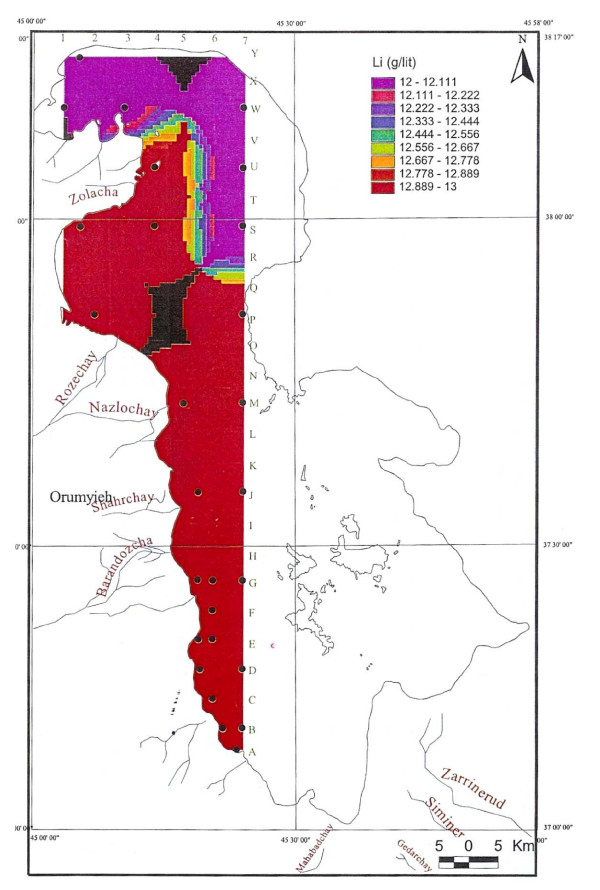
Lithium concentration in the top 0.5 m of Urmia Lake in fall.

### Winter sampling

From 115 stations, 216 samples were collected from the surface (-0.5 m), middle (-5 m) and bottom samples (-7.5 m.). K was measured in all samples but only 22 samples were analyzed for Mg, Ca, Na, Br and Li.

Potassium in the surface samples averages 1.5 g/lit (figure 9.2). Compared to the fall samples (figure 9.1), K has increased from 1.44 to 1.62 g/lit. The winter concentration of K, in both surface and middle samples of the winter is higher than in the other two seasons. This may probably is due to mirabilite formation in the cold weather in south than north and also shallower water in the south. This lowers the Na and SO4 contents and raising the mole percent of K. The boundary of K variation clearly corresponds to the Kalantary causeway position. The causeway may have prevented the circulation of K between north and south in the winter stronger than what it showed for fall (figures 9.2 and 10.2). Both samples show the same pattern of lower K levels in the north increasing toward south.

Magnesium, with concentrations of 5.8 to 6.3 g/lit, is highly enriched in the north. The high- concentration areas occupy most of the lake from mid-north to north, and may result from weathering of ulterabasic rocks in the Nazloo basin, but are diluted in the far south due to higher flow from the south. Therefore, Mg is more uniform with slight variation near the river deltas (figure 14.2).

Sodium shows a great variation due to water fluctuation. It is enriched in the north part of the lake, but higher in the middle, and depleted in the far south of the lake. This results from more water charge from the south (figure 12.2).

Calcium shows depletion in the north part of the lake, and enrichment in the south half. The concentrations are clearly separated by the causeway, and resulted from differing Ca inputs by two the largest rivers (Zarineh and Simineh) flowing from south to the lake, compared to the small rivers feeding the lake from north (figure 13.2).

### Spring sampling

In the spring season, 186 samples were analyzed for K and 23 for Mg, Ca, Na, Br and Li. Potassium in the surface and middle samples varied from 1.45 to 1.54 g/lit, with a similar distribution pattern. Some low-concentration sampling cells near river deltas are caused by dilution from higher inflow especially in the mid-center portion of the lake. No preferential elevated zone was observed in the lake due to rapid circulation, accelerated by huge amounts of water that flow from all the sides to the lake during the spring (figures 9.3 and 10.3). This preferential pattern is not clear in the bottom samples due to insufficient data (figures 9.3 and 11.3).

Calcium varies between 3.3 to 3.5 g/lit, and is concentrated in the western- and mid-center banks due to higher water inflow. There are strong variations in the far north and far south parts of the lake because of rapid water circulation (figure 13.3).

Sodium in the spring stage varies between 81.9 to 86.9 g/lit. Overall, sodium at (83.57 g/lit) is lower compared to 89 g/lit in the winter and 87.11 g/lit in the fall. This is due to the increase of water fluctuation in the spring (figures 12.1, 12.2, 12.3).

Magnesium varies between 2.32 to 3.04 g/lit, and is concentrated in the south with an irregular pattern, and even greater variation in the north (figure 14.3).

## Conclusion

As prepared, maps show geochemical distribution patterns that are not very uniform, but do show that the differences between seasonal lateral and vertical data are not significant, thus implying good mixing through out the lake. This may be accelerated by river inflows from all around the lake, and not from a particular side. The maximum depth of Urmia Lake is about 9 meters, though only in a limited area, thus Urmia Lake is classified as a shallow lake with large area. There are slight differences between the north and south parts of the lake in all seasons, and due to its shallowness, it has a quick seasonal turnover.

Mirabilite precipitates mainly in the shallow areas of the southern part of the lake in the cold weather of fall and winter. Salt forms only in the summer in pools and along the lake banks. Salt, on the other hand, usually starts dissolving very quickly when the fall rains begin. Mirabilite formation is observed mainly south of causeway in the lake. In the north, it is reported only in some unharvested salt pools until the weather gets cold. The exact condition of mirabilite formation is not well established here. It is very similar to salt, but after exposure to air and dryness it converts to a very soft, white amorphous powder called thenardite. Salt remains relatively unchanged upon becoming dry. Mirabilite also is bitter compared to normal salt and easily recognized in salt pools wherever is formed.

Big rivers carrying fresh water flow from the south into the lake, so there is a high turbidity flow from south to north. The Agichay River, entering the lake from northeast, brings in salty water. Because of mixing, there is a very small variation in salinity between the two ends of the lake. It also looks as if the higher turbidity coming from south has deposited a large amount of sediments behind the causeway dike (Sedighian, I. and Barzgar, F. 2002). There is a distinct vertical temperature change at about 1.5 m below the surface in deep areas of the lake, but no significant differences in areas of shallower depth.

## Results

Surface samples taken during the three seasons show somehow higher concentration of K in the south. This indicates that most of K may have been carried in solution by large rivers entering the lake from the south (12–15 ppm for Zarineh and Simineh rivers compared to 5–7 ppm for Aji-chay in the north), [9], and may also be influenced by higher evaporation. Spring shows a more even distribution of chemistry than the other seasons, due to rapid circulation and mixing the water, and shows no preferred area of concentration. In the middle samples (5 m>depth>0.5 m), K increases in the south up to 1.57 g/lit in winter and decreases in the spring to 1.55 g/lit. In the winter, the bottom samples also show higher K concentration. Therefore, the highest concentration of K is found in the winter. It appears that the effect of the causeway is clearer in this season possibly due to less water or slower circulation.

In general no significant differences in K concentration are observed at different depths. Over all, the change in concentration is in between 1.4 to 1.6 g/lit indicating a rather homogeneous K distribution. But, it looks like the Kalantary causeway affects the pattern of K along the road, which is clear in fall and winter with increased K to the south of the causeway.

Salts, over all, are lower in Urmia Lake except for halite compared to its twin sister (Great Salt Lake) in Utah, USA, particularly in K. The very low K content of Urmia Lake may make K recovery uneconomical in comparison to Utah's Great Salt Lake.

Calcium is depleted in the north and enriched in the south half, being clearly separated by the causeway. The elevated calcium level in the south part of the lake is attributed to the two largest, calcium-rich rivers (Zarineh roud and Simineh roud) flowing into the lake from the south, compared to the small, lower-calcium rivers feeding the lake from the north and from the west. Magnesium is slightly higher at the lake's mid-center, and may be derived from ulterabasic rocks by the Nazloo River.

Regardless of small differences between major elements during various seasons, Urmia Lake seems compositionally to be very homogenous. This may be due to the strong currents in the lake caused by the water flowing into the lake from both north and south. But due to higher evaporation and higher temperature at top, the amount of Na and Cl is higher in the surface. The temperature of the lake water is always higher in upper 1.5 m., especially in the spring and summer seasons and therefore the upper water is lighter than the water below this level, which prevents rapid vertical movement from surface to bottom.

Since trace elements such as bromine and lithium are not enriched in the lake, their differences can not be strongly differentiated in different depths, and they look homogenous trough out the Lake. Common salt is the only mineral which is exploited economically at this time. Mg, K, and Li may be evaluated for exploitation in the future if suitable technologies are employed.

In the winter, in many places around the banks of the lake, sodium sulphate (thenardite and mirabilite) crystallizes when the lake water temperature reaches below 8°C. If harvested and processed, mirabilite could be considered as a potential economical product of the lake.
